# Investigation of the Microbial Diversity in the *Oryza sativa* Cultivation Environment and Artificial Transplantation of Microorganisms to Improve Sustainable Mycobiota

**DOI:** 10.3390/jof10060412

**Published:** 2024-06-06

**Authors:** Yeu-Ching Shi, Yu-Juan Zheng, Yi-Ching Lin, Cheng-Hao Huang, Tang-Long Shen, Yu-Chia Hsu, Bao-Hong Lee

**Affiliations:** 1 Department of Food Sciences, National Chiayi University, Chiayi 60004, Taiwan; jasmineycs70@gmail.com; 2 Department of Horticultural Sciences, National Chiayi University, Chiayi 60004, Taiwan; ny97321@gmail.com (Y.-J.Z.); q20020311@gmail.com (Y.-C.L.); 3 Department of Food Safety/Hygiene and Risk Management, College of Medicine, National Cheng Kung University, Tainan 701401, Taiwan; doodle29068@gmail.com; 4 Department of Plant Pathology and Microbiology, National Taiwan University, Taipei 10617, Taiwan; shentl@ntu.edu.tw; 5 Department of Agronomy, National Chiayi University, Chiayi 60004, Taiwan; hsuychia@mail.ncyu.edu.tw

**Keywords:** rice straw, soil, *Oryza sativa*, mycobiota, sustainable agriculture, phytohormones

## Abstract

Rice straw is not easy to decompose, it takes a long time to compost, and the anaerobic bacteria involved in the decomposition process produce a large amount of carbon dioxide (CO_2_), indicating that applications for rice straw need to be developed. Recycling rice straw in agricultural crops is an opportunity to increase the sustainability of grain production. Several studies have shown that the probiotic population gradually decreases in the soil, leading to an increased risk of plant diseases and decreased biomass yield. Because the microorganisms in the soil are related to the growth of plants, when the soil microbial community is imbalanced it seriously affects plant growth. We investigated the feasibility of using composted rice stalks to artificially cultivate microorganisms obtained from the *Oryza sativa*-planted environment for analyzing the mycobiota and evaluating applications for sustainable agriculture. Microbes obtained from the water-submerged part (group-A) and soil part (group-B) of *O. sativa* were cultured in an artificial medium, and the microbial diversity was analyzed with internal transcribed spacer sequencing. Paddy field soil was mixed with fermented paddy straw compost, and the microbes obtained from the soil used for *O. sativa* planting were designated as group-C. The paddy fields transplanted with artificially cultured microbes from group-A were designated as group-D and those from group-B were designated as group-E. We found that fungi and yeasts can be cultured in groups-A and -B. These microbes altered the soil mycobiota in the paddy fields after transplantation in groups-D and -E compared to groups-A and -B. Development in *O. sativa* post treatment with microbial transplantation was observed in the groups-D and -E compared to group-C. These results showed that artificially cultured microorganisms could be efficiently transplanted into the soil and improve the mycobiota. Phytohormones were involved in improving *O. sativa* growth and rice yield via the submerged part-derived microbial medium (group-D) or the soil part-derived microbial medium (group-E) treatments. Collectively, these fungi and yeasts may be applied in microbial transplantation via rice straw fermentation to repair soil mycobiota imbalances, facilitating plant growth and sustainable agriculture. These fungi and yeasts may be applied in microbial transplantation to repair soil mycobiota imbalances and sustainable agriculture.

## 1. Introduction

Macromolecular carbohydrates contained in rice stalks can be decomposed and utilized by many microorganisms. Therefore, many studies have developed rice stalks to treat swine manure digested effluent [1]. Most of the rice straw is not properly utilized but is disposed of by burning, discarding, and landfilling, and this improper disposal can cause unexpected consequences for the soil environment and for air quality. In recent years, the digestion mediated by microbial fermentation has been applied to rice straw degradation for sustainable application and a clean approach, including compound production and transformation [2]. On the other hand, *Bacillus ligniniphilus* L1 and *Mucor indicus* were used in bioethanol production via rice straw degradation [3,4], biogas production was generated with rice straw through anaerobic digestion [5], and hydrogen recovery from rice straw was achieved via fermentation in a bioelectrochemical system [6].

Soil state is affected by pesticide use, chemical fertilizers, pollution, and extreme climate, and the diversity of microbial species in the soil is also destroyed [7]. Evidence has suggested that by 2050 the microbial species in the soil may have decreased by more than 30% [8]. Because the probiotics in the soil are related to the growth of plants, when the soil microbial imbalance is more serious, it may threaten grain yield as well as agricultural sustainability. The rhizosphere is an important plant environment that regulates nutrient transformation and absorption and thus directly affects plant growth. A study investigating the root environment of peppers planted in greenhouses found that although there was no significant difference in organic carbon, the metabolites and microbial community differed between the roots and soil [9]. A study has found that microorganisms have protective effects on plants, and specific microorganisms can reduce the absorption of heavy metals in the soil by *O. sativa* [10]. Plant roots contain a group of microorganisms called plant-growth-promoting rhizobacteria, which can regulate the production and synthesis of phenolic compounds in plants and can secrete antibacterial substances to inhibit the growth of pathogenic bacteria [11]. Additionally, soil microorganisms synthesize phytohormones that enhance plant stress tolerance and thus improve resistance to harsh environments (including salinization, temperature, heavy metals, and drought) [12].

In addition to isolating microorganisms from soil to explore their physiological functions and growth regulation on plants, past research has also isolated microorganisms from wastewater and evaluated the Bacillus subtilis isolated from wastewater for improving soil quality to enhance rice yield. In this study, a four-week soil inoculation and anaerobic incubation revealed that B. subtilis positively impacts carbohydrate and nitrogen mineralization in long-term paddy soil. Therefore, inoculating Bacillus subtilis is recommended as an environmentally friendly method to enhance soil fertility and reduce nitrogen immobilization. Further research on applying microbial inoculation in rice plants is needed to consolidate the preliminary results [13]. However, the relationship between the microbial community and environmental climate change has been investigated, but whether the altered microbes can be restored via transplantation remains unclear. One currently feasible approach involves improving the living environment of *O. sativa* by transplanting healthy microorganisms. In a previous study, soil transplantation between different geographic climate regions was performed to investigate changes in microbial communities [14].

Additionally, several studies have reported that yeast and fungi are important for root development and growth promotion in *O. sativa* [15,16,17,18]. These studies suggested that transplantation with soil yeast/fungi may help improve *O. sativa* growth. Rice straw could be used as a nutrient source for many fungi and yeasts; hence, we evaluated the mycobiota difference between the soil (underground) and submerged (above ground) parts of *O. sativa* for treating *O. sativa* with rice straw in this study.

## 2. Materials and Methods

### 2.1. Compost Preparation

According to a previous study [19], sewage sludge was added with rice straw for compost preparation; we prepared compost with modifications by omitting sewage sludge. Briefly, dry *O. sativa* straw (20 kg; 48.2%) was pre-moistened by soaking in water for 2 days and then softened until 60% water content was achieved. The *O. sativa* straw was mixed with paddy soil (40 kg; 48.2%), urea (540 g; 1.3%), ammonium sulfate (540 g; 1.3%), and CaCO_3_ (430 g; 1.0%) to ferment for 30 days (environmental temperature about 37–40 °C), and the compost was turned every 10 days.

### 2.2. Microbial Cultivation

Different microorganisms were obtained from paddy fields, including submerged- (above ground) and soil parts (underground) of planted *O. sativa*, which were cultured with a formula containing deionized water (10 L), molasses (50 g), rice bean powder (10 g), fermented compost (100 g) that was sterilized (121 °C, 30 min), and the submerged (above ground) (as group-A) or soil part (underground) (as group-B) of the *O. sativa* plant harvested from paddy fields (50 g). After cultivation (7–10 days, 25 °C), the microbes from the cultured medium were collected via centrifugation (3000 rpm, 15 min), and these microbe suspensions were inoculated into planted *O. sativa*.

### 2.3. Microbial Transplantation into Planted O. sativa

The *O. sativa* (species: No. Tainung-81 from hybridization of *O. sativa* Taikeng 2× *O. sativa* Chianungyu 902036) was seeded and was grown until it reached 45–50 cm plant height and was then transplanted into 2 kg of rice straw compost (soaked in 3 L sterilized water) for group-C. Moreover, these *O. sativa* plants were transplanted into compost and treated with microbial transplantation (inoculation; 80 g microbial medium/kg compost) with submerged-part (from group-A) and soil-part (group-B) donor microbes for group-D and group-E, respectively. The microbial transplantation was realized in the *O. sativa*-planted environment after nursery transplantation three times (1 time every 10 days). The rhizosphere microbes were investigated with ITS analysis after 40 days.

### 2.4. Assay for Mycobiota with Next-Generation Sequencing

Microbes were collected via centrifugation, and DNA extraction was carried out referring to the manufacturer instructions for the Bioworld Fungi/Yeast Genomic DNA Isolation Kit (Thermo Fisher Scientific Inc., Waltham, MA, USA). For the rapid purification of fungal DNA, DNA was submitted to sensitive, chemical-free (phenol and chloroform) spin-column treatment. The DNA quality and quantity were checked with a NanoDrop spectrophotometer (Thermo Fisher Scientific, Waltham, MA, USA). The internal transcribed spacer (ITS) regions of DNA were amplified with fungi primers ITS1: 5′-GCTGCGTTCTTCATCGATGC-3′ and ITS5: 5′- GGAAGTAAAAGTCGTAACAAGG-3′. The PCR products were high-throughput sequenced with the Illumina MiSeq platform and a Miseq Reagent kit v3 (Illumina, Inc., Santiago, CA, USA). The DNA cluster was defined according to Amplicon Sequence Variants (ASVs) with a threshold of 97% sequence identity via the unite database (v8.2; 2020.02.20).

### 2.5. Assay for Phytohormones

After freeze-drying 0.2 g of soil, two small steel balls and 1 mL of extract solution (50% acetonitrile in water) were added and then sonicated and homogenized in an ice-water bath (repeating the cycle three times) for metabolite extraction. Centrifugation at 14,000× *g* for 15 min at 4 °C was realized. Then, 900 μL of the supernatant was evaporated under a gentle stream of nitrogen until dryness was achieved and was then reconstitute in 90 μL of 10% ACN/H_2_O (*v*/*v*). The samples (85 μL) were analyzed using UHPLC-MS/MS (EXIONLC System, Waters ACQUITY UPLC CSH C18 column (150 × 2.1 mm, 1.7 μm, Waters, Milford, MA, USA)). The mobile phase A was 0.01% formic acid in water, and the mobile phase B was 0.01% formic acid in acetonitrile. The column temperature was maintained at 50 °C. The auto-sampler temperature was set at 4 °C, and the injection volume was 5 μL. Each phytohormone standard solution (at different concentrations) was injected into the API source of the mass spectrometer. SCIEX Analyst Work Station software (Version 1.6.3) and Sciex MultiQuant™ 3.0.3 were used for data acquisition and analysis

### 2.6. Statistical Analysis

All data were presented as mean ± SD. The one-way analysis of variance (ANOVA) and Duncan’s test (*p* < 0.05) were used to evaluate the statistical significance (SAS Inc., Cary, NC, USA) for biomass, yield (plant height, panicles, panicle weight, panicle length, and seed numbers), and phytohormone levels. The assay for alpha-diversity was investigated using Shannon and Simpson analysis.

## 3. Results

### 3.1. The Investigation of Mycobiota in Different Samples

This study first aimed to understand the bacterial community in the *O. sativa* cultivation environment, including the original irrigation water, original soil, and original rhizosphere soil. On the other hand, we also attempt to explore the cultivable microorganisms using broth, including those from cultivated irrigation water, cultivated soil, and cultivated rhizosphere soil. As shown in Supplementary Figure S1, the bacterial abundance in irrigation water, soil, and rhizosphere soil was evaluated in original samples compared to cultivated samples. From the results, it is evident that alpha-diversity in cultivated irrigation water, cultivated soil, and cultivated rhizosphere soil are all lower than those in the original irrigation water, original soil, and original rhizosphere soil (Supplementary Figure S2). The beta-diversity was also different in the original irrigation water vs. the cultivated irrigation water; the original soil vs. the cultivated soil; and the original rhizosphere soil vs. the cultivated rhizosphere soil (Supplementary Figure S3). The results also reveal that only a minority of microorganisms can be cultured from all samples as seen in Venn diagrams of the analysis for the bacterial OTU (Supplementary Figure S4).

In the original irrigation water, original soil, and original rhizosphere soil, the majorities of bacterial species remain unclear. In the top-10 results, it is observed that after cultivation, the predominant microorganism in the original irrigation water is Lactobacillus, while in the original soil and original rhizosphere soil, the main bacteria are Lactococcus and Enterobacter, respectively (Supplementary Figure S5). The main biomarkers are presented in Supplementary Figure S6.

**Figure 1:**
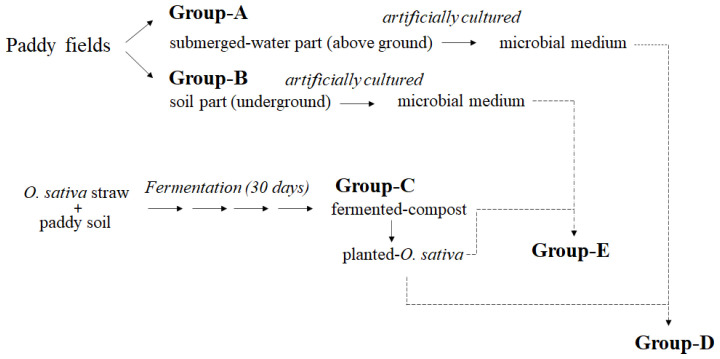
The flowchart for experiments.

**Figure 2:**
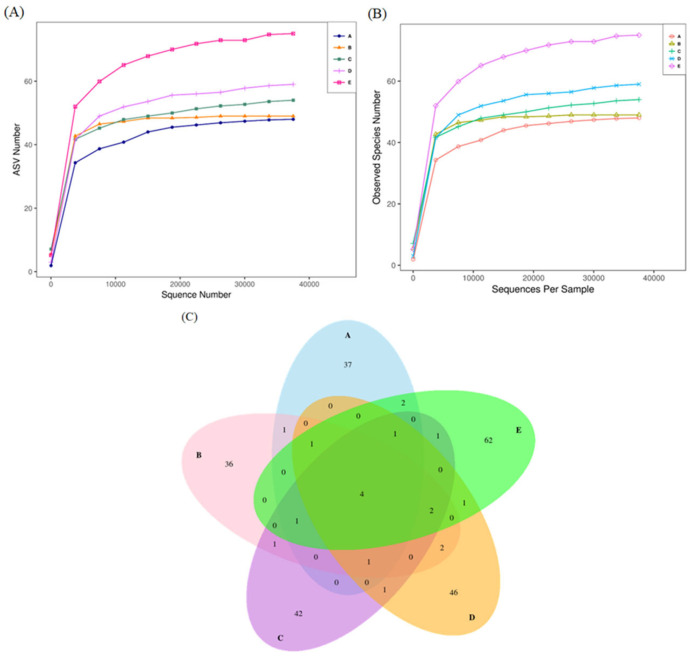
Alpha diversity of microbial communities. (**A**) The abscissa represents the sequenced number, and the vertical axis represents the number of ASVs that can be constructed based on the sequenced number. (**B**) The rarefaction curve with the extracted sequence and the corresponding number of species to represent the diversity of species. (**C**) Venn diagrams of the analysis for the microbial ASV in each group. Group-A: submerged part-derived microbial medium; Group-B: soil part-derived microbial medium; Group-C: *O. sativa* planted in control compost; Group-D: *O. sativa* planted in compost transplanted with submerged part-derived microbial medium; Group-E: *O. sativa* planted in compost transplanted with soil part-derived microbial medium.

Since Lactobacillus, Lactococcus, and Enterobacter are common bacteria with documented impacts on the environment and organisms, this study further investigates the mycobiota in *O. sativa* cultivation environments. In recent years, many research findings have emphasized the relationship between soil fungi and plant host growth [9,10]. Although soil fungi play crucial roles in plants and cultivation environments, fungal diversity is gradually diminishing [8]. Therefore, establishing a novel method to maintain or enhance soil fungal communities is the focus of this study. It evaluates the effects of cultivating soil fungi and transplantation methods on O. sativa. A flowchart of microbial transplantation into *O. sativa* soil is shown in Figure 1. The fungal diversity in groups-A, -B, -C, -D, and -E was evaluated. Figure 2A,B show that numerous microorganisms can be cultured based on the ASV number and the number of observed species. Figure 2C shows Venn diagrams after cluster analysis.

**Figure 3:**
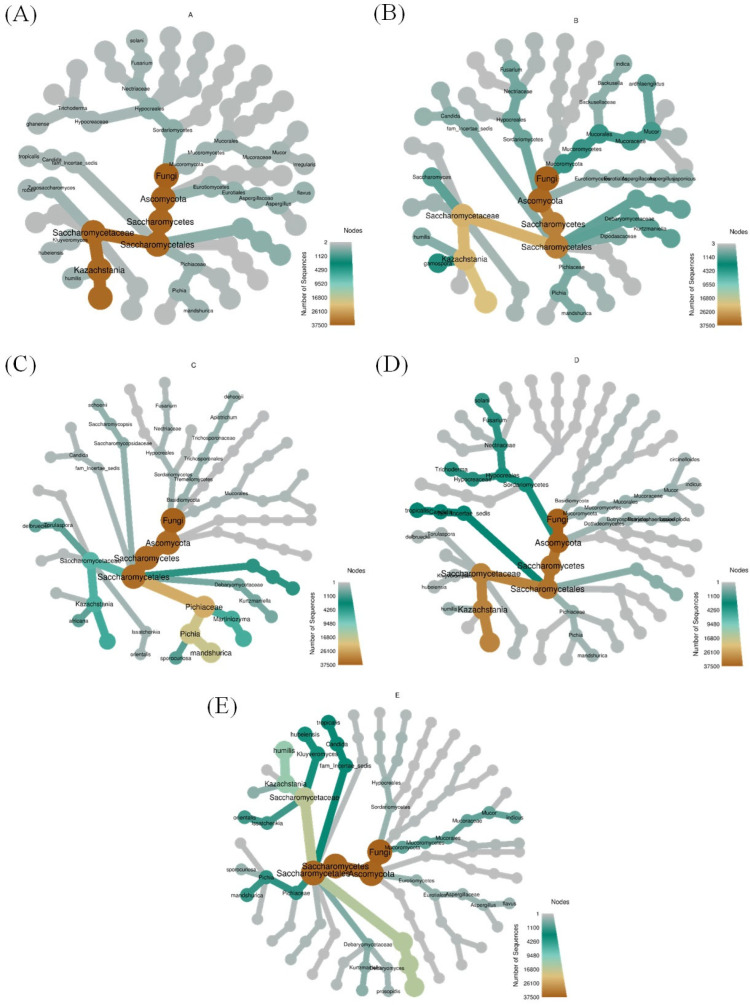
The heat-tree analysis according to bacteria mycobiota in each group. (**A**) Group-A: submerged part-derived microbial medium; (**B**) Group-B: soil part-derived microbial medium; (**C**) Group-C: *O. sativa* planted in control compost; (**D**) Group-D: *O. sativa* planted in compost transplanted with submerged part-derived microbial medium; and (**E**) Group-E: *O. sativa* planted in compost transplanted with soil part-derived microbial medium.

The specific fungal OTU numbers were 37, 36, 42, 46, and 62 in groups-A, -B, -C, -D, and -E, respectively; however, only 4 OTUs were found in all groups. The heat-tree plot shows the top microbes in each group, as shown in Figure 3. The abundance of the top microbes is marked by size, branch thickness, and color. We found that the differential taxonomic tree showed significant changes in taxon abundance in each group. From the kingdom to species levels, Ascomycota, Saccharomycetes, and Saccharomycetales were found in all groups. Similar results were found in groups-A and -B, and similar microbial classification was observed in groups-C, -D, and -E.

**Figure 4:**
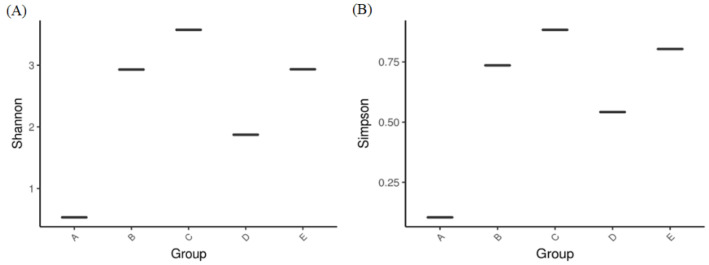
(**A**) The Shannon and (**B**) Simpson indexes of alpha-diversity obtained from analysis of bacteria mycobiota between groups. Group-A: submerged part-derived microbial medium; Group-B: soil part-derived microbial medium; Group-C: *O. sativa* planted in control compost; Group-D: *O. sativa* planted in compost transplanted with submerged part-derived microbial medium; and Group-E: *O. sativa* planted in compost transplanted with soil part-derived microbial medium.

**Figure 5:**
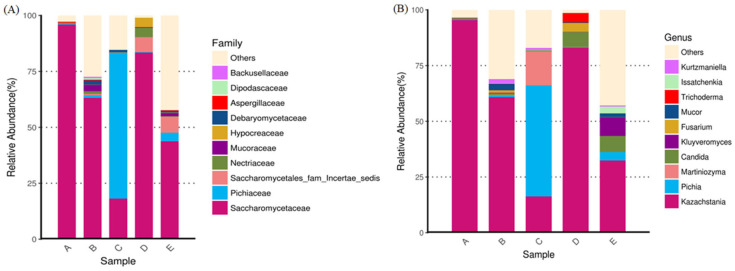
The top-10 abundance from taxa analysis of mycobiota composition for (**A**) family and (**B**) genus. Group-A: submerged part-derived microbial medium; Group-B: soil part-derived microbial medium; Group-C: *O. sativa* planted in control compost; Group-D: *O. sativa* planted in compost transplanted with submerged part-derived microbial medium; and Group-E: *O. sativa* planted in compost transplanted with soil part-derived microbial medium.

**Figure 6:**
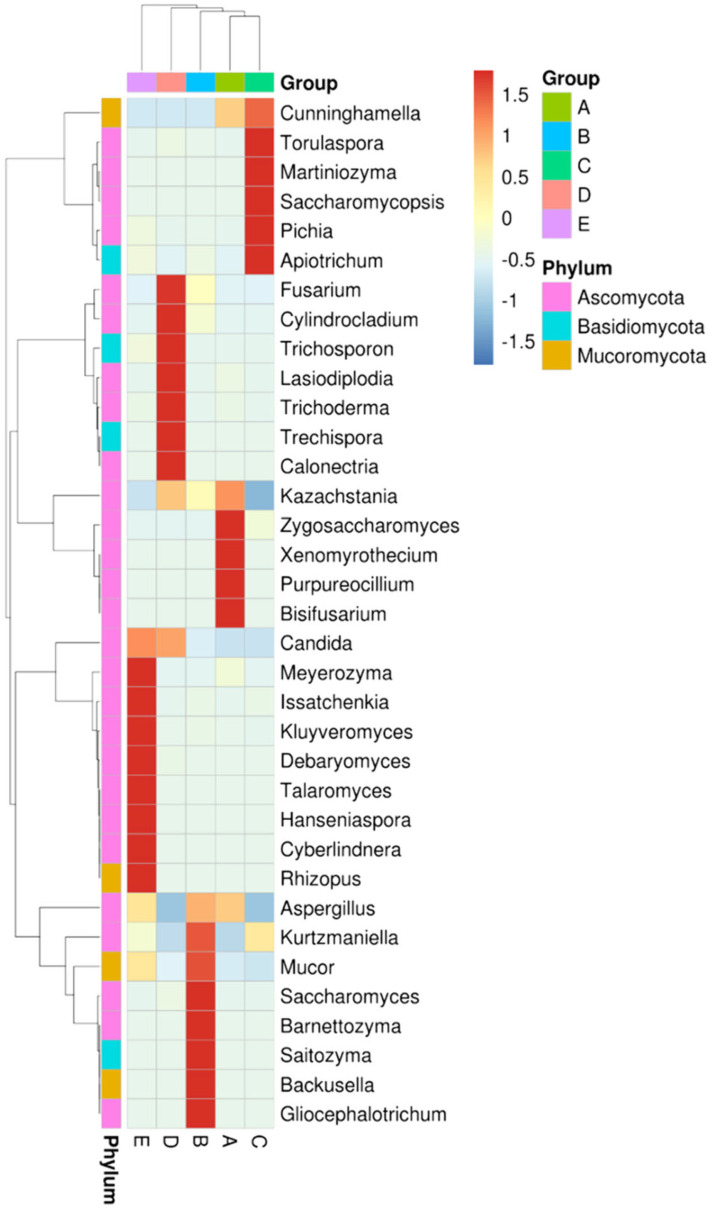
Taxa analysis of mycobiota composition in each group with heatmap. Group-A: submerged part-derived microbial medium; Group-B: soil part-derived microbial medium; Group-C: *O. sativa* planted in control compost; Group-D: *O. sativa* planted in compost transplanted with submerged part-derived microbial medium; and Group-E: *O. sativa* planted in compost transplanted with soil part-derived microbial medium.

Figure 4 shows the Shannon and Simpson indices in all groups for the evaluation of alpha diversity, indicating that the alpha diversity of group-A was similar to that of group-D, while that of group B was similar to that of group-E. As shown in Figure 5, mycobiota analysis at the family level revealed that Saccharomycetaceae was abundant in both groups-A and -B, but the microbial diversity was more complex in group B than in group-A. Additionally, Pichiaceae was the top microbe in the control compost in group-C. When O. sativa was planted on control compost treated with submerged part-derived and soil part-derived microbial medium, the mycobiota changed in groups-D and -E; Saccharomycetaceae became the dominant microbes, and the level of Saccharomycetales_fam_Incertae_sedis was also elevated. At the genus level, Kazachstania was the dominant yeast in Saccharomycetaceae, which contains over 40 distinct species. Figure 6 shows the specific microbes in diverse groups. Cunninghamella, Torulaspora, Martiniozyma, Saccharomycopsis, Pichia, and Apiotrichum were found in group-C; Zygosaccharomyces, Xenomyrothecium, Purpureocillium, and Bisifusarium were found in group-A; Kurtzmaniella, Mucor, Saccharomyces, Barnettozyma, Saitozyma, Backusella, and Gliocephalotrichum were found in group-B; Fusarium, Cylindrocladium, Trichosporon, Lasiodiplodia, Trichoderma, Trechispora, and Calonectria were found in group-D; and Meyerozyma, Issatchenkia, Kluyveromyces, Debaryomyces, Talaromyces, Hanseniaspora, Cyberlindnera, and Rhizopus were found in group-E.

### 3.2. Root Development of O. sativa Treated with Microbial Transplantation

**Figure 7:**
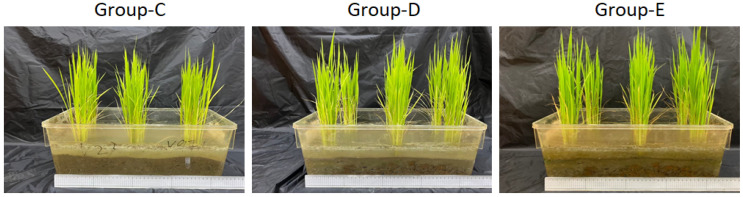
The growth of *O. sativa* treated with microbial transplantation.

In this study, *O. sativa* was planted in compost transplanted with submerged part-derived microbial medium (group-D) or soil part-derived microbial medium (group-E), and the growth condition was evaluated comparing to the *O. sativa* planted in control compost (group-C). As shown in Table 1 and Figure 7, microbial supplementation could markedly promote growth of *O. sativa* after transplantation, as well as increasing rice biomass and yields, because phytohormones are related to rice yield, and many soil microorganisms have also been reported to produce and secrete phytohormone. Therefore, we further analyzed the phytohormone content in the soil with different treatments with submerged part-derived microbial medium (group-D) or soil part-derived microbial medium (group-E), and the results are shown in Table 2. 1-aminocyclopropanecarboxylic acid, indole-3-carboxaldehyde, indole-3-acetic acid (IAA), 3-indolebutyric acid, N6-isopentenyladenosine, and salicylic acid content in group-D and group-E were significantly higher than those in group-C. These findings suggest that yeast may produce different phytohormones that improve *O. sativa* growth and rice yield.

## 4. Discussion

Intensive rice cultivation to meet food demand generates large amounts of rice straw, approximately 600–1000 metric tons as agricultural waste annually [20]. This rice straw has been developed into different products, including biochar, but the benefits of these products for sustainable net zero production are not as expected. The decomposition of rice straw is an attractive solution to open-field burning but this strategy takes 60–90 days to obtain mature compost. In the rice field ecosystem, after the straw is buried in the soil, the decomposed small molecules can provide a rich source of nutrients for methanogens and can cause the generation and emission of CH_4_ in the rice field [21]. Therefore, the applications for the rapid decomposition of rice straw and the regulation of carbon footprint via rice straw were investigated [22,23]. 

In addition to maintaining the nutrient status of the soil and providing a healthy environment for plants, soil fungi make a great contribution to carbon fixation [24]. Fungi do not have chloroplasts and cannot perform photosynthesis like plants, so it is impossible for them to directly convert carbon dioxide in the air into carbohydrates. However, after hundreds of millions of years of evolution, many species of fungi and plants have developed a close “symbiosis”. In this relationship, fungi intertwine with plant roots to form a network structure (mycorrhiza). Fungi help plants absorb water and phosphorus and other mineral nutrients, and plants deliver some carbohydrates to fungi for energy. Therefore, when plants absorb carbon dioxide on the ground to produce sugars, fungi also quietly provide assistance underground and store the carbon dioxide absorbed by plants in the soil [25]. Tackling the massive crisis of biodiversity loss may start in a very small way. A study showed that if the microbial community of bacteria, fungi, algae, and archaea can be restored, the growth rate of plants can be increased by an average of 64%. Scientists pointed out that this discovery can help protect various microorganisms in the soil and restore ecosystems [26].

In addition to studying the soil ecosystem, it is an important topic to try to restore the state of the soil through soil transplants. Obtaining soil from a healthy forest with high microbial diversity and transplanting it to land with low microbial diversity to restore the ecosystem is a highly anticipated strategy for the future.

Using anaerobic digestion technology to treat agricultural waste can not only reduce the amount of agricultural waste, but also convert it into clean energy to meet the needs of the times. Therefore, the recycling application of rice straw is a subject of much attention. For example, rice straw biochar was used to inhibit the generation of polycyclic aromatic hydrocarbons in an integrated rice and crab co-culture system [27]. The reduction in soil carbon concentration will lead to a serious imbalance of soil microbiota, which will interfere with the growth of crops, which will not only negatively affect the development of sustainable agriculture but also potentially endanger global food security [28,29]. Fungi and yeast play an important role in rice fields. If these microorganisms can be cultivated and use rice straw as a nutrient source, it will help to develop technologies for artificially transplanting microorganisms to improve soil mycobiota and characteristics [30,31]. Soil microbial transplantation is a method that can be used to rectify soil microbial imbalance. It is based on the premise that these complex microorganisms can be cultured in a low-cost and efficient manner through special culturing methods. Artificial cultivation of natural bacterial community composition has been explored, but perfecting fungi cultivation for utilization remains unclear [32].

In this study, fungi and yeasts were cultured in soil medium and applied to the soil for *O. sativa* cultivation, and the changes in microorganisms were analyzed. The results showed that many microorganisms that promote plant growth can be cultured and transplanted into soil. Among these microbes, yeast has potential for accelerating plant growth [19]. Yeast can be used as an effective bio-tool for promoting tolerance in plants against drought stress [33]. As most soil microorganisms are still difficult to culture artificially, DNA sequencing of these microorganisms is difficult, and their functions cannot be confirmed; however, they play numerous roles in the adjustment of ecosystem functions [19]. Although numerous types of microorganisms exist in the soil, only a small number of specific microorganisms coexist with plants. These microbes live in symbiosis with plants and are found in the subterranean rhizosphere and the upper phyllosphere of plants. They exist as endophytes within the plant, epiphytes that attach to plant surfaces, and around roots in the surrounding soil [19]. Intensive planting and the use of pesticides substantially reduce the diversity and population of phytosymbiotic soil microorganisms. These reduced microorganisms may benefit plant health, development, growth, and biomass yield. Therefore, restoring soil with dysbiosis to a good state through specific methods to increase beneficial bacteria and reduce phytopathogenic bacteria should improve the yield and disease resistance of cultivated plants [34].

*Cunninghamella* is an endophytic fungus from soil or organic substates that has frequently been found in saprotrophs, soil, dung, and other organic substrates. This microbe has potential for producing IAA in culture media, which can increase chlorophyll, carotenoid, glucose, abscisic acid, fructose, and sucrose content in plants, reduce hydrogen peroxide, and inhibit protein and lipid metabolism to regulate various plant growth stresses [35]. *Torulaspora* is found in the natural environments (soil, plants, and fruits) and also has potential for promoting plant growth [36]. One study has reported that *Saccharomycopsis*-derived nonvolatile metabolites, organic acids, and chitinase act against phytopathogenic infections [37]. The yeast *Pichia* may produce plant hormones and promote root development [38]. Insights into the *Apiotrichum* and *Saitozyma* communities of fungal root endophytes have revealed that these microbes can elevate phytohormone levels in tomato plants [39]. *Zygosaccharomyces* has been used as a bio-control agent for phytopathogens [40]. Moreover, previous studies have reported that *Purpureocillium*, *Barnettozym,* and *Cylindrocladium* can function as plant-growth-promoting agents via IAA production [41,42,43]. The protection and plant growth promotion effects of *Mucor* have also been reported [44]. *Trichoderma* could promote plant growth and reduce the cost of pathogen elimination during cultivation [45,46]. The soil yeast *Meyerozyma* was found to promote the growth of multiple plants by increasing phosphate solubilization [47]. The anaerobic fungus *Kluyveromyces* has been found to hydrolyze lignocellulose and produce free sugars and organic acids to promote plant growth [48]. The benefits of *Talaromyces* in biocontrol and plant growth promotion were attributed to protection against *Ganoderma* basal stem rot disease [49]. The potential of the yeast *Debaryomyces* and *Rhizopus* for amelioration of arsenic stress in plants has been evaluated [50].

Many types of yeast exist in the soil, and the benefits of these yeasts to plants have been reported in the above studies, including pathogenic bacteria inhibition in the soil [50,51,52,53] as well as stimulation of IAA production to promote plant root growth [54]. IAA is an auxin-class phytohormone which plays an important role in plants, including cell division and expansion, cell differentiation, and fruit development [55]. A study has reported that IAA-produced bacteria were implicated in promoting the growth of *O. sativa* [56]. The phytohormone salicylic acid is a well-known signal molecule regulating plant immunity and enhanced defense gene expression for disease resistance in *O. sativa* [57]. An association between beneficial yeast in soil and salicylic acid for accelerating *O. sativa* growth has been reported [58]. A rhizobacterium (*Pseudomonas aeruginosa*) could improve systemic acquired resistance in beans by producing salicylic acid [59]. Moreover, several benefit microorganisms have been found to generate indole-3-carboxaldehyde [60]; this compound has potential for inhibiting pathogens [61]. 3-indolebutyric acid is likely to be converted into IAA in a process similar to fatty acid β-oxidation. Multiple lines of evidence suggest that 3-indolebutyric acid-derived auxin promotes root hair expansion [62]. Yeast is an important microbe in the culture medium in this study, as it assists in the colonization of host plants by producing and secreting IAA that stimulate filament growth (pseudohyphae). However, the high-efficiency microorganism strains for IAA, salichlic acid, indole-3-carboxaldehyde, and 3-indolebutyric acid production need to be isolated for application in plant growth in the future. Therefore, our study aimed to develop a strategy for soil microbial cultivation using soil microbe transplantation. Soil microbes are essential for maintaining life-supporting ecosystem services; however, investigation of how these microbes are preserved in extreme environments is important. We found that various microorganisms were cultured and applied in *O. sativa* for cleaning rice straw, including *Fusarium, Cylindrocladium, Trichosporon, Lasiodiplodia, Trichoderma, Trechispora, Calonectria, Meyerozyma, Issatchenkia, Kluyveromyces, Debaryomyces, Talaromyces, Hanseniaspora, Cyberlindnera,* and *Rhizopus*.

## 5. Conclusions

Straw is one of the main byproducts of the agricultural production process. As the world’s largest producer of grain, China is also the world’s largest producer of straw. A rice farming environment can effectively promote sustainable agricultural development, and it is designed based on the ecological characteristics of rice. Soil state is affected by pollution and extreme climate year by year, the diversity of microbial species in the soil is also being destroyed. The microbial species in the soil may decrease by more than 30%; because the probiotics in the soil are related to the growth of plants, when the soil microbial imbalance is more serious, it may affect the yield of food. This study is concerned with such topics; we cultured microorganisms by separating microorganisms from above and below the ground of rice plants. In addition, important fungi/yeasts in the rice cultivation environment were discussed, and these potential probiotics were used in a transplantation method to explore the enhancement of rice growth and rice yield, and our results showed that there were different important fungi/yeasts in the aboveground and underground environments in the rice plant context, and the content of plant hormones in the soil was related to these microorganisms, and we hope that, in the future, the soil with its imbalance of bacteria can be repaired and crop yield can be improved by using microbial transplantation.

## Figures and Tables

**Table 1:** The characteristic evaluation of *O. sativa* for biomass and yield after microbial transplantation.

Parameters	Plant Height (cm)	Panicles/Plant (Numbers)	Panicle Weight (g)	Panicle Length (cm)	Seed/Panicle (Numbers)
Group-C	84.6 ± 7.8 ^a^	7.1 ± 2.4 ^b^	9.11 ± 3.7	15. 2± 3.1 ^a^	14.6 ± 4.1 ^b^
Group-D	80.1 ± 10.4 ^a^	9.2 ± 3.3 ^ab^	13.11 ± 2.2	17.5 ± 4.2 ^a^	23.3 ± 5.7 ^ab^
Group-E	82.4 ± 8.2 ^a^	10.3 ± 1.6 ^a^	12.76 ± 2.6	16.3 ± 3.5 ^a^	29.5 ± 3.9 ^a^

Data are shown as mean ± SD (n = 6). Significant difference was assigned with *p* < 0.05 with various superscript letters.

**Table 2:** The evaluation of metabolites for phytohormones in soil with *O. sativa*.

Metabolites *	Group C	Group D	Group E
	Concentration (nM)		
1-Aminocyclopropanecarboxylic acid	31.7 ± 6.3 ^b^	43.7 ± 8.2 ^ab^	53.1 ± 13 ^a^
Indole-3-carboxaldehyde	14.5 ± 3.2 ^b^	28.2 ± 14.7 ^a^	31.4 ± 4.3 ^a^
Indole-3-acetic acid	2.1 ± 0.2 ^b^	16.3 ± 3.5 ^a^	21.9 ± 7.1 ^a^
3-Indolebutyric acid	0.17 ± 0.06 ^b^	0.55 ± 0.28 ^a^	0.44 ± 0.03 ^a^
N6-(delta 2-Isopentenyl)-adenine	0.04 ± 0.00	0.06 ± 0.00	0.06 ± 0.03
Kinetin	0.17 ± 0.03	0.16 ± 0.01	0.18 ± 0.01
trans-Zeatin	0.02 ± 0.01	0.04 ± 0.01	0.04 ± 0.01
cis-Zeatin	0.07 ± 0.01	0.10 ± 0.09	0.11 ± 0.05
N6-isopentenyladenosine	0.03 ± 0.01 ^b^	0.14 ± 0.09 ^a^	0.14 ± 0.00 ^a^
Salicylic acid	30.5 ± 5.8 ^c^	179.8 ± 26.8 ^b^	290.3 ± 92.8 ^a^
(±)-Jasmonic acid	2.5 ± 0.8	2.2 ± 0.3	3.3 ± 0.8
Dihydrojasmonic Acid	0.59 ± 0.14	0.78 ± 0.41	1.34 ± 0.25
N-((-)-jasmonoyl)-S-isoleucine	0.13 ± 0.04	0.21 ± 0.01	0.23 ± 0.01
Methyl 3-indolylacetate	N/D	N/D	N/D
Methyl salicylate	N/D	N/D	N/D
DL-Dihydrozeatin	N/D	N/D	N/D
Methyl jasmonate	N/D	N/D	N/D
trans-Zeatin-riboside	N/D	N/D	N/D
(+)-Abscisic acid	N/D	N/D	N/D
Gibberellin A7	N/D	N/D	N/D
Gibberellin A4	N/D	N/D	N/D
Gibberellin A3	N/D	N/D	N/D
Gibberellin A1	N/D	N/D	N/D

* Data are shown as mean ± SD (n = 3). Significant difference was assigned with *p* < 0.05 (various superscript letters). N/D: not detected.

## Data Availability

The original contributions presented in the study are included in the article/Supplementary Materials, further inquiries can be directed to the corresponding author.

## Supplementary Materials

The following supporting information can be downloaded at: https://www.mdpi.com/article/10.3390/jof10060412/s1, Supplementary Figure S1. The bacterial abondance in (A) irrigation water, (B) soil, and (C) rhizosphere soil were evaluated in original samples comparing to cultivated-samples. Supplementary Figure S2. The alpha-diversity of (A) irrigation water, (B) soil, and (C) rhizosphere soil in original samples compared to the cultured-samples by Shannon statistics. Supplementary Figure S3. The beta-diversity of (A) irrigation water, (B) soil, and (C) rhizosphere soil in original samples compared to the cultured-samples by PCA statistics. Supplementary Figure S4. The Venn diagrams of analysis for the bacterial OUT. (A) irrigation water, (B) soil, and (C) rhizosphere soil in original samples compared to the cultured-samples. Supplementary Figure S5. The major bacterial OUT appeared by Top-10. (A) irrigation water, (B) soil, and (C) rhizosphere soil in original samples compared to the cultured-samples. Supplementary Figure S6. The main biomarkers for (A) irrigation water, (B) soil, and (C) rhizosphere soil in original samples compared to the cultured-samples.

## References

[1] Zheng H., Tang F., Lin Y., Xu Z., Xie Z., Tian J. (2022). Solid-state anaerobic digestion of rice straw pretreated with swine manure digested effluent. J. Clean. Prod..

[2] Cañon C., Sanchez N., Cobo M. (2022). Sustainable production of ethyl levulinate by levulinic acid esterification obtained from Colombian rice straw. J. Clean. Prod..

[3] Nazar M., Xu L., Ullah M.W., Moradian J.M., Wang Y., Sethuparhy S., Iqbal B., Nawaz M.Z., Zhu D. (2022). Biological delignification of rice straw using laccase from *Bacillus ligniniphilus* L1 for bioethanol production: A clean approach for agro-biomass utilization. J. Clean. Prod..

[4] Molaverdi M., Mirmohamadsadeghi S., Karimi K., Aghbashlo M., Tabatabaei M. (2022). Efficient ethanol production from rice straw through cellulose restructuring and high solids loading fermentation by *Mucor indicus*. J. Clean. Prod..

[5] Li H., Wang C., Chen X., Xiong L., Guo H., Yao S., Wang M., Chen X., Huang C. (2022). Anaerobic digestion of rice straw pretreatment liquor without detoxification for continuous biogas production using a 100 L internal circulation reactor. J. Clean. Prod..

[6] Singh N.K., Singh R. (2023). Hydrogen recovery cascade from pretreated rice straw and its fermentative residuals using step-up potential-based sulfate reducing bacteria-bioelectrochemical system. J. Clean. Prod..

[7] Walder F., Schmid M.W., Riedo J., Valzano-Held A.Y., Banerjee S., Buchi L., Bucheli T.D., van der Heijden M.G.A. (2022). Soil microbiome signatures are associated with pesticide residues in arable landscapes. Soil Biol. Biochem..

[8] Rahman N.S.N.A., Hamid N.W.A., Nadarajah K. (2021). Effects of abiotic stress on soil microbiome. Int. J. Mol. Sci..

[9] Song Y., Li X., Yao S., Yang X., Jiang X. (2020). Correlations between soil metabolomics and bacterial community structures in the pepper rhizosphere under plastic greenhouse cultivation. Sci. Total Environ..

[10] Treesubsuntorn C., Dhurakit P., Khaksar G., Thiravetyan P. (2018). Effect of microorganisms on reducing cadmium uptake and toxicity in rice (*Oryza sativa* L.). Environ. Sci. Pollut. Res..

[11] Korenblum E., Aharoni A. (2019). Phytobiome metabolism: Beneficial soil microbes steer crop plants secondary metabolism. Pest Manag. Sci..

[12] Egamberdieva D., Wirth S.J., Alqarawi A.A., Abd-Allah E.F., Hashem A. (2017). Phytohormones and beneficial microbes: Essential components for plants to balance stress and fitness. Front. Microbiol..

[13] Toan N.S., Nguyen T.D.P., Thu T.T.N., Lim D.T., Dong P.D., Gia N.T., Khoo K.S., Chew K.W., Show P.L. (2021). Soil mineralization as effects of plant growth promoting bacteria isolated from microalgae in wastewater and rice straw application in a long-term paddy rice in Central Viet Nam. Environ. Technol. Innov..

[14] Liang Y., Jiang Y., Wang F., Wen C., Deng Y., Xue K., Qin Y., Yang Y., Wu L., Zhou J. (2015). Long-term soil transplant regulating climate change with latitude significantly alters microbial temporal turnover. ISME J..

[15] Khaskheli M., Wu L., Chen G., Chen L., Hussain S., Song D., Liu S., Feng G. (2020). Isolation and characterization of root-associated bacterial endophytes and their biocontrol potential against major fungal phytopathogens of rice (*Oryza sativa* L.). Pathogens.

[16] Amprayn K.O., Rose M.T., Kecskes M., Pereg L., Nguyen H.T., Kennedy I.R. (2012). Plant growth promoting characteristics of soil yeast (*Candida tropicalis* HY) and its effectiveness for promoting rice growth. Appl. Soil Ecol..

[17] Lu H., Qi X., Ur Rahman S., Qiao D., Li P., Han Y., Zhao Z. (2021). Rice physiological response with *Bacillus subtilis* and *Saccharomyces cerevisiae* inoculation into soil under reclaimed water-fresh water combined irrigation. Water.

[18] Yergeau E., Bell T.H., Champagne J., Maynard C., Tardif S., Tremblay J., Greer C.W. (2015). Transplanting soil microbiomes leads to lasting effects on Willow growth, but not on the rhizosphere microbiome. Front. Microbiol..

[19] Roca-Pérez L., Martínez C., Marcilla P., Boluda R. (2009). Composting rice straw with sewage sludge and compost effects on the soil-plant system. Chemosphere.

[20] Foong S.Y., Chan Y.H., Chin B.L.F., Lock S.S.M., Yee C.Y., Yiin C.L., Peng W., Lam S.S. (2022). Production of biochar from rice straw and its application for wastewater remediation—An overview. Bioresor. Technol..

[21] Qin X., Li Y., Wang H., Liu C., Li J., Wan Y., Gao Q., Fan F., Liao Y. (2016). Long-term effect of biochar application on yield-scaled greenhouse gas emissions in a rice paddy cropping system: A four-year case study in south China. Sci. Total Environ..

[22] Sarma S., Patel N., Patel A., Desai C., Sharma S., Dedania S., Rudaykiya D.M., Vishwakarma A.S., Vahora S., Narra M. (2022). Rapid decomposition of rice straw by application of novel microbial consortium and study its microbial community dynamics. World J. Microbiol. Biotechnol..

[23] Qin X., Lu Y., Wan Y., Wang B., Nie J., Li Y., Liao Y. (2023). Rice straw application improves yield marginally and increases carbon footprint of double cropping paddy rice (*Oryza sativa* L.). Field Crop. Res..

[24] Hannula S.E., Morriën E. (2022). Will fungi solve the carbon dilemma?. Geoderma.

[25] Charters M.D., Sait S., Field K.J. (2020). Aphid herbivory drives asymmetry in carbon for nutrient exchange between plants and an arbuscular mycorrhizal fungus. Curr. Biol..

[26] Averill C., Anthony M.A., Baldrian P., Finkbeiner F., van den Hoogen J., Kiers T., Kohout P., Hirt E., Smithe G.R., Crowther T.W. (2022). Defending earth’s terrestrial microbiome. Nat. Microbiol..

[27] Sun N., Yu S., Cai Z., Liu J., Wang T., Qi B., Wang Z., Wang S., Yang A., Zhu G. (2022). Inhibition of polycyclic aromatic hydrocarbon (PAHs) release from sediments in an integrated rice and crab coculture system by rice straw biochar. J. Clean. Prod..

[28] Sritongon N., Sarin P., Theerakulpisut P., Riddech N. (2022). The effect of salinity on soil chemical characteristics, enzyme activity and bacterial community composition in rice rhizospheres in Northeastern Thailand. Sci. Rep..

[29] Chandra P., Khippal A.K., Prajapat K., Barman A., Singh G., Rai A.K., Ahlawat O.P., Verma R.P.S., Kumari K., Singh G. (2023). Influence of tillage and residue management practices on productivity, sustainability, and soil biological properties of rice-barley cropping systems in indo-gangetic plain of India. Front. Microbiol..

[30] Tang H., Li C., Xiao X., Shi L., Cheng K., Wen L., Li Y. (2020). Effects of shor-term manure nitrogen in put on soil microbial community structure and diversity in a double-cropping paddy field of southern China. Sci. Rep..

[31] Haiming T., Xiaoping X., Chao L., Xiaochen P., Kaikai C., Weiyan L., Ke W. (2020). Microbial carbon source utilization in tice rhizosphere and nonrhizosphere soils with short-term manure N input rate in paddy field. Sci. Rep..

[32] Agrahari R.K., Singh P., Koyama H., Panda S.K. (2020). Plant-microbe interactions for sustainable agriculture in the post-genomic era. Curr. Genom..

[33] Alzandi A.A., Naguib D.M. (2022). Effect of yeast application on soil health and root metabolic status of corn seedlings under drought stress. Arch. Microbiol..

[34] Guerra C.A., Delgado-Baquerizo M., Duarte E., Marigliano O., Gorgen C., Maestre F.T., Eisenhauer N. (2021). Global projections of the soil microbiome in the Anthropocene. Glob. Ecol. Biogeogr..

[35] Wang Z., Chen Z., Kowalchuk G.A., Xu Z., Fu X., Kuramae E.E. (2021). Succession of the resident soil microbial community in response to periodic inoculations. Appl. Environ. Microbiol..

[36] Kazerooni E., Maharachchikumbura S., Al-Sadi A., Rashid U., Kim I.D., Kang S.M., Lee I.J. (2022). Effects of the rhizosphere fungus *Cunninghamella bertholletiae* on the *Solanum lycopersicum* response to diverse abiotic stresses. Int. J. Mol. Sci..

[37] De Oliveira T.B., Junior R.B., Silva L.G., Rosa-Magri M.M. (2019). Rhizosphere yeast *Torulaspora globose* with plant growth promotion traits and improvement of the development of tomato seedling. s under greenhouse conditions. Afric. J. Agric. Res..

[38] Junker K., Chailyan A., Hesselbart A., Forster J., Wendland J. (2019). Multi-omics characterization of the necrotrophic mycoparasite *Saccharomycopsis schoenii*. PLoS Pathog..

[39] Giri R., Sharma R.K. (2020). Fungal pretreatment of lignocellulosic biomass for the production of plant hormone by *Pichia fermentans* under submerged conditions. Bioresour. Bioprocess..

[40] Manzotti A., Bergna A., Burow M., Jorgensen H.J.L., Cernava T., Berg G., Collinge D.B., Jensen B. (2020). Insights into the community structure and lifestyle of the fungal root endophytes of tomato by combining amplicon sequencing and isolation approaches with phytohormone profiling. FEMS Microbiol. Ecol..

[41] Ferraz P., Cassio F., Lucas C. (2019). Potential of yeasts as biocontrol agents of the phytopathogen causing cacao witches’ broom disease: Is microbial warfare a solution?. Front. Microbiol..

[42] Carla Baron N., de Souza Pollo A., Rigobelo E.C. (2020). *Purpureocillium lilacinum* and *Metarhizium marquandii* as plant growth-promoting fungi. PeerJ.

[43] Fu S.F., Sun P.F., Lu H.Y., Wei J.Y., Xiao H.S., Fang W.T., Cheng B.Y., Chou J.Y. (2016). Plant growth-promoting traits of yeasts isolated from the phyllosphere and rhizosphere of *Drosera spatulate* Lab. Fungal Biol..

[44] Yang J., Ye T., Liu G., Xu X., Zheng Y., Wang W. (2019). Synthesis and bioactivity of indoleacetic acid-carbendazim and its effects on *Cylindrocladium parasiticum*. Pestic. Biochem. Physiol..

[45] Nartey L.K., Pu Q., Zhu W., Zhang S., Li J., Yao Y., Hu X. (2021). Antagonistic and plant growth promotion effects of *Mucor moelleri*, a potential biocontrol agent. Microbiol. Res..

[46] Hermosa R., Viterbo A., Chet I., Monte E. (2012). Plant-beneficial effects of *Trichoderma* and of its genes. Microbiology.

[47] Hermosa R., Rubio M.B., Cardoza R.E., Nicolas C., Monte E., Gutierrez S. (2013). The contribution of *Trichoderma* to balancing the costs of plant growth and defense. Int. Microbiol..

[48] Nakayan P., Hameed A., Singh S., Young L.S., Hung M.H., Young C.C. (2013). Phosphate-solubilizing soil yeast *Meyerozyma guilliermondii* CC1 improves maize (*Zea mays* L.) productivity and minimizes requisite chemical fertilization. Plant Soil.

[49] Hillman E.T., Li M., Hooker C.A., Englaender J.A., Wheeldon I., Solomon K.V. (2021). Hydrolysis of lignocellulose by anaerobic fungi produces free sugars and organic acids for two-stage fine chemical production with *Kluyveromyces marxianus*. Biotechnol. Prog..

[50] Goh Y.K., Marzuki N.F., Tuan Pa T.N.F., Goh T.K., Kee Z.S., Goh Y.K., Yusof M.T., Vujanovic V., Goh K.J. (2020). Biocontrol and plant-growth-promoting traits of *Talaromyces apiculatus* and *Clonostachys rosea* consortium against Ganoderma basal stem rot disease of oil palm. Microorganisms.

[51] Kaur J., Anand V., Srivastava S., Bist V., Tripathi P., Naseem M., Nand S., Khare P., Srivastava P.K., Bisht S. (2020). Yeast strain *Debaryomyces hansenii* for amelioration of arsenic stress in rice. Ecotoxicol. Environ. Saf..

[52] Srivastava P.K., Shenoy B.D., Gupta M., Vaish A., Mannan S., Singh N., Teware S.K., Tripathi R.D. (2012). Stimulatory effects of arsenic-tolerant soil fungi on plant growth promotion and soil properties. Microbes Envrion..

[53] El-Tarabily K.A., Sivasithamparam K. (2006). Potential of yeasts as biocontrol agents of soil-borne fungal plant pathogens and as plant growth promoters. Mycoscience.

[54] Rao R.P., Hunter A., Kashpur O., Normanly J. (2020). Aberrant synthesis of indole 3-acetic acid in *Saccharomyces cerevisiae* triggers morphogenic transition, a virulence trait of pathogenic fungi. Genetics.

[55] Rayle D.L., Cleland R.E. (1992). The acid growth theory of auxin-induced cell elongation is alive and well. Plant Physiol..

[56] Susilowati D.N., Riyanti E.I., Setyowati M., Nulya K. (2018). Indole-3-acetic acid producing bacteria and its application on the growth of rice. AIP Conf. Proc..

[57] Liang B., Wang H., Yang C., Wang L., Qi L., Guo Z., Chen X. (2022). Salicylic acid is required for broad-spectrum disease resistance in rice. Int. J. Mol. Sci..

[58] Bowya T., Balachandar D. (2020). Harnessing PGPR inoculation through exogenous foliar application of salicylic acid and microbial extracts for improving rice growth. J. Basic Microbiol..

[59] De Meyer G., Capieau K., Audenaert K., Buchala A., Métraux J.P., Höfte M. (1999). Nanogram amounts of salicylic acid produced by the rhizobacterium *Pseudomonas aeruginosa* 7NSK2 activate the systemic acquired resistance pathway in bean. Mol. Plant Microb. Interact..

[60] Teng Y., Ren Y., Sayed M., Hu X., Lei C., Kumar A., Hutchins E., Mu J., Deng Z., Luo C. (2018). Plant-derived exosomal microRNAs shape the gut microbiota. Cell Host Microbe.

[61] Que Y., Huang D., Gong S., Zhang X., Yuan B., Xue M., Shi W., Zeng F., Liu M., Chen T. (2022). Indole-3-carboxylic acid from the endophytic fungus *Lasiodiplodia pseudotheobromae* LPS-1 as a synergist enhancing the antagonism of jasmonic acid against *Blumeria graminis* on wheat. Front. Cell. Infect. Microbiol..

[62] Růžička K., Strader L.C., Bailly A., Yang H., Blakeslee J., Langowski L., Nejedla E., Fujita H., Itoh H., Syono K. (2010). Arabidopsis PIS1 encodes the ABCG37 transporter of auxinic compounds including the auxin precursor indole-3-butyric acid. Proc. Natl. Acad. Sci. USA.

