# Supplementary material for: Investigation of the Microbial Diversity in the Oryza sativa Cultivation Environment and Artificial Transplantation of Microorganisms to Improve Sustainable Mycobiota
Source: J Fungi (Basel). 2024 Jun 6;10(6):412. doi: 10.3390/jof10060412 (PMC11205129; doi:10.3390/jof10060412)
Supplement: Supplementary file 1 [file jof-10-00412-s001.zip › jof-3023338-supplementary.pdf]

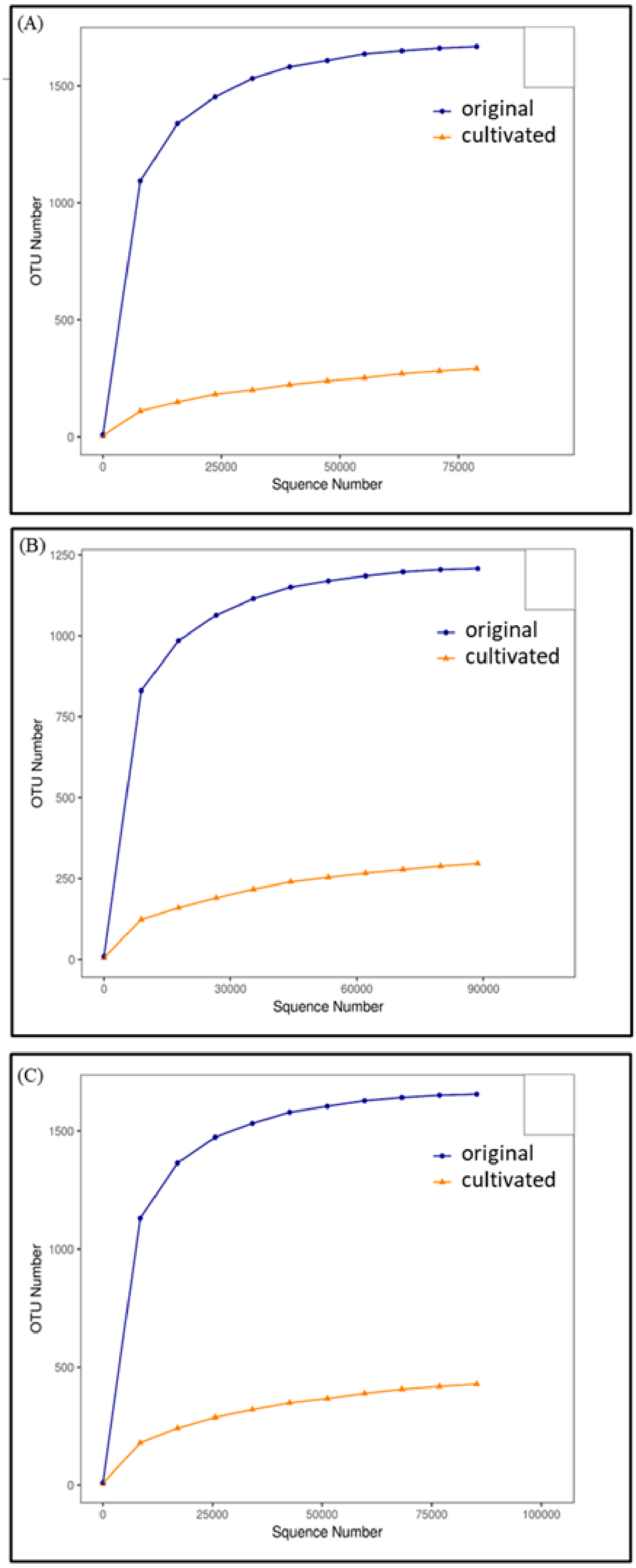

Supplementary Figure S1. The bacterial abondance in (A) irrigation water, (B) soil, and (C) rhizosphere soil were evaluated in original samples comparing to cultivated-samples.

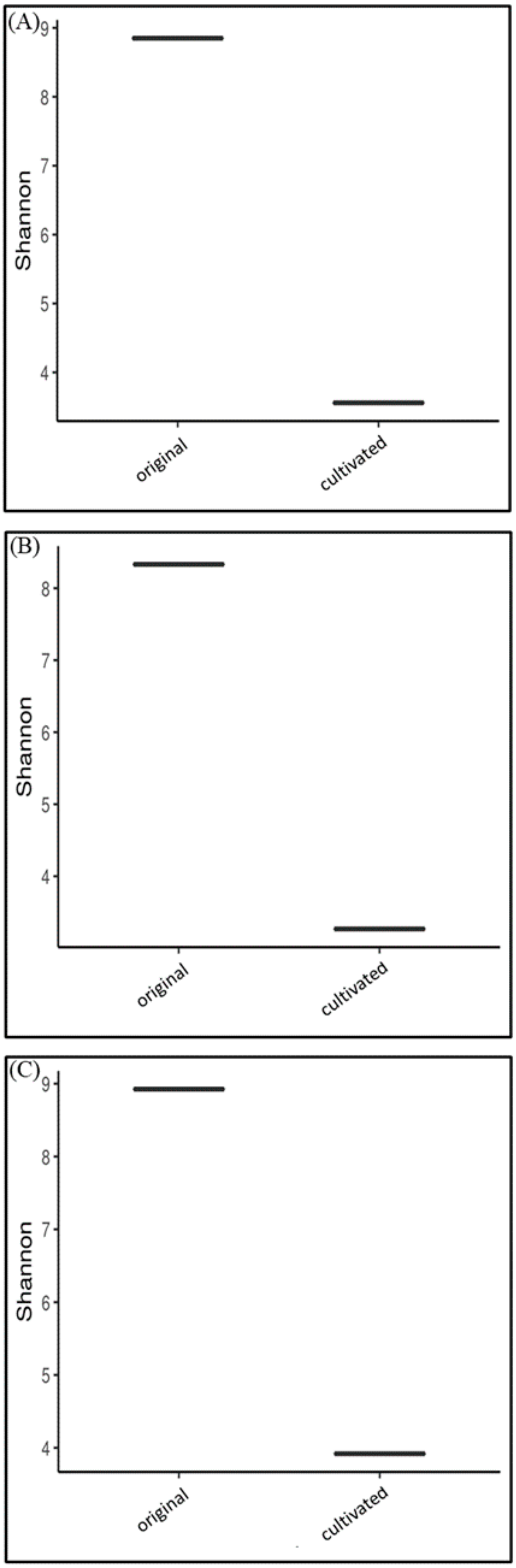

Supplementary Figure S2. The alpha-diversity of (A) irrigation water, (B) soil, and (C) rhizosphere soil in original samples compared to the cultured-samples by Shannon statistics.

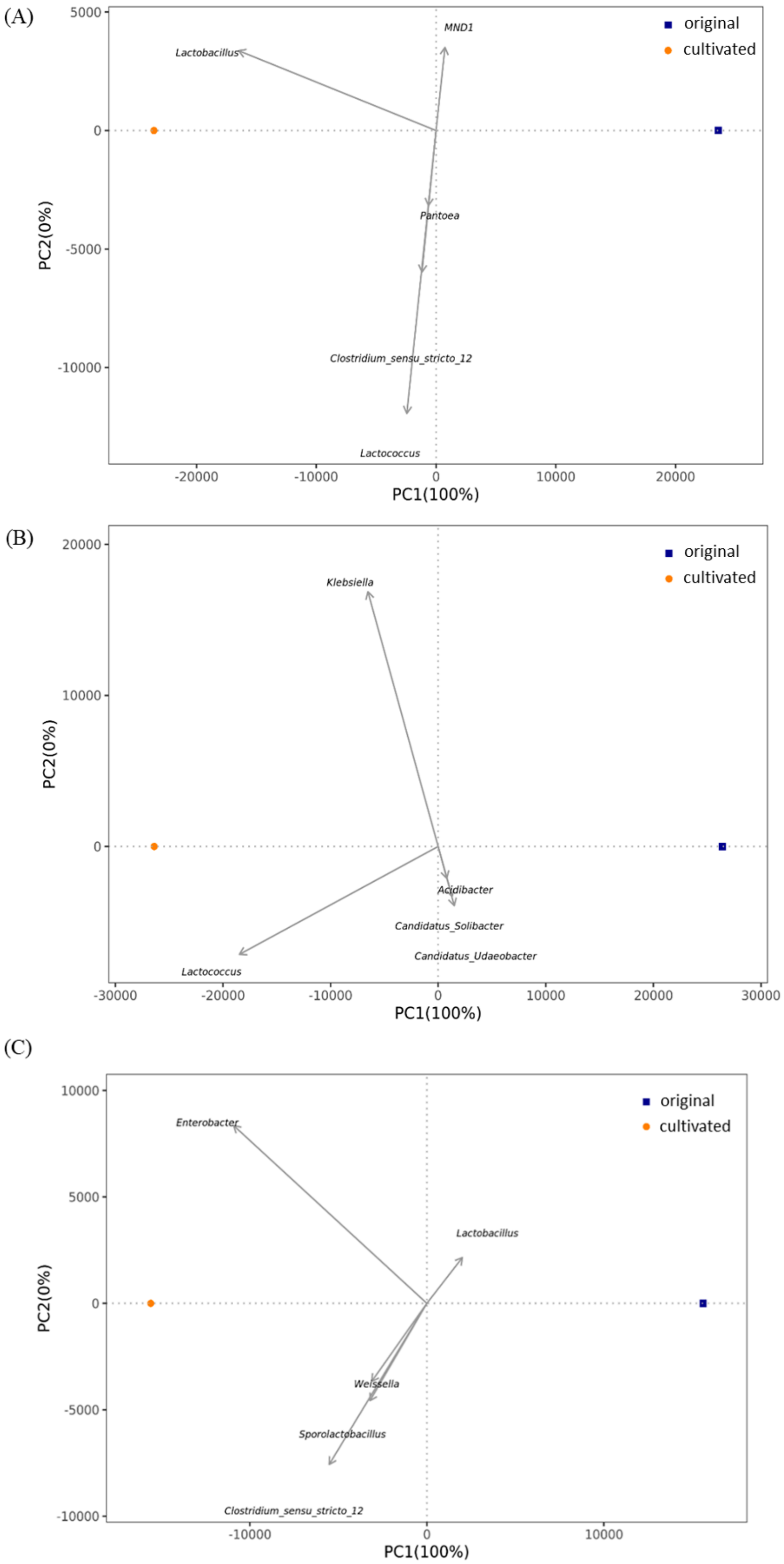

Supplementary Figure S3. The beta-diversity of (A) irrigation water, (B) soil, and (C) rhizosphere soil in original samples compared to the cultured-samples by PCA statistics.

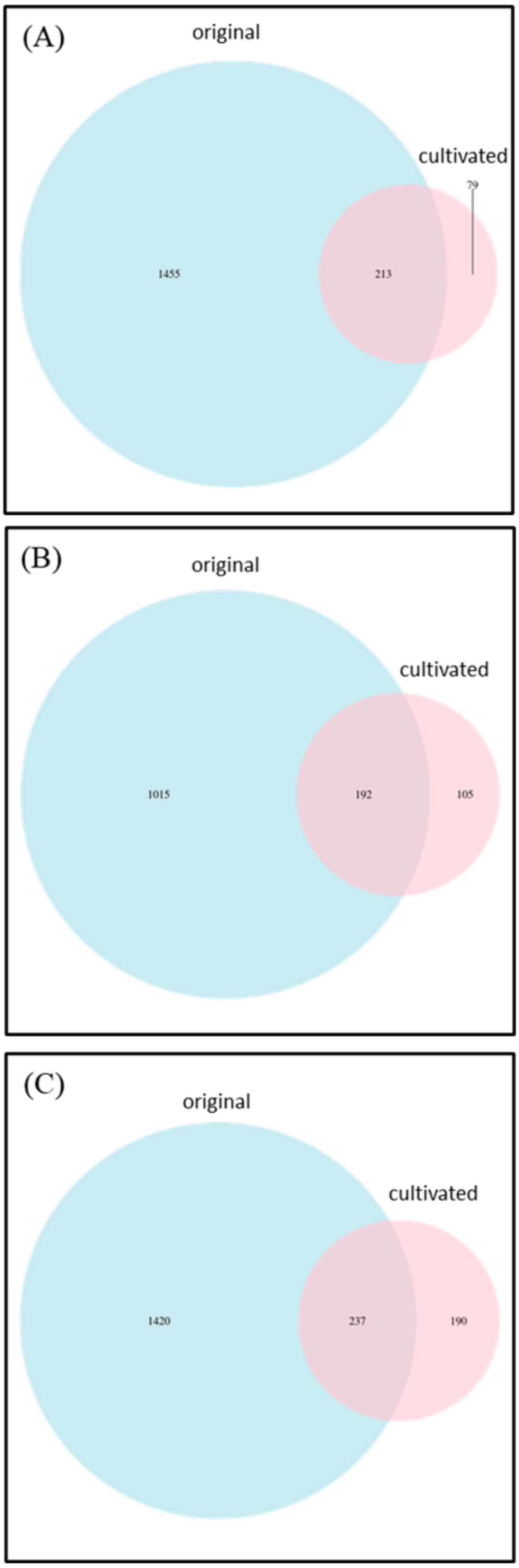

Supplementary Figure S4. The Venn diagrams of analysis for the bacterial OUT. (A) irrigation water, (B) soil, and (C) rhizosphere soil in original samples compared to the cultured-samples

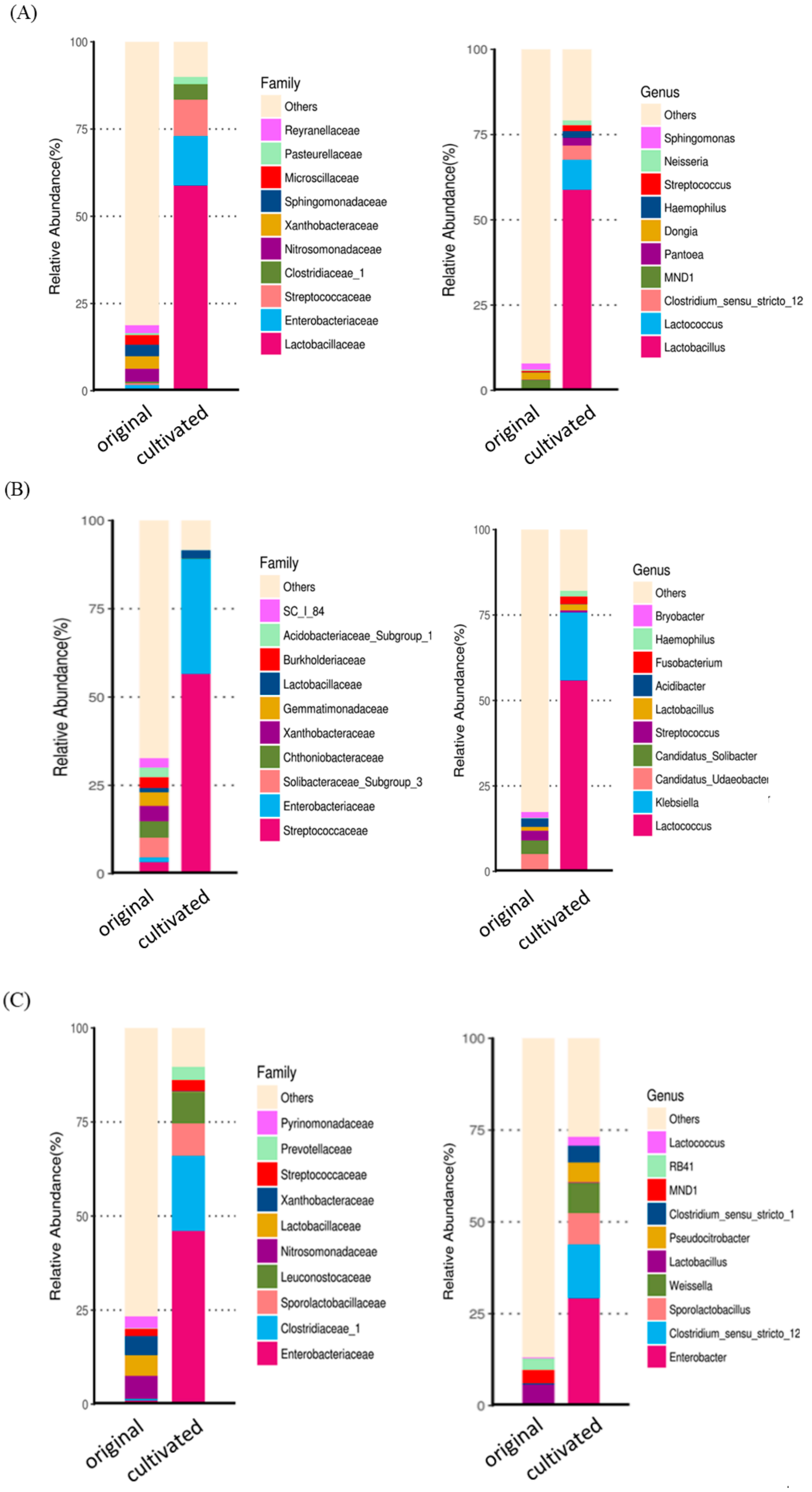

Supplementary Figure S5. The major bacterial OUT appeared by Top-10. (A) irrigation water, (B) soil, and (C) rhizosphere soil in original samples compared to the cultured-samples.

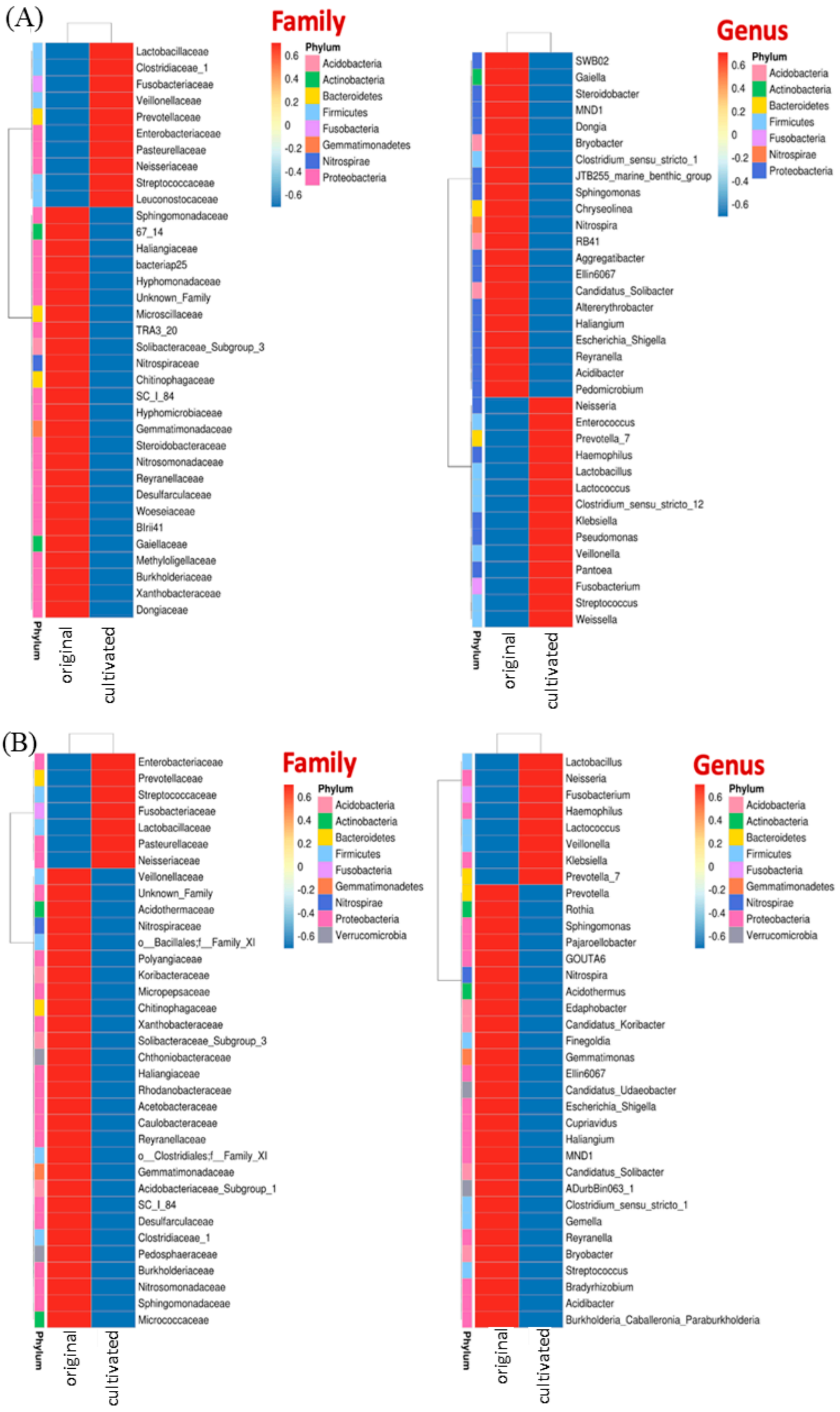

(A)
Family
Phylum
0.6
0.4
0.2
0
-0.2
-0.4
-0.6
Acidobacteria
Actinobacteria
Bacteroidetes
Firmicutes
Fusobacteria
Gemmatimonadetes
Nitrospirae
Proteobacteria
Lactobacillaceae
Clostridiaceae_1
Fusobacteriaceae
Veillonellaceae
Prevotellaceae
Enterobacteriaceae
Pasteurellaceae
Neisseriaceae
Streptococcaceae
Leuconostocaceae
Sphingomonadaceae
67_14
Haliangiaceae
bacteriap25
Hyphomonadaceae
Unknown_Family
Microscillaceae
TRA3_20
Solibacteraceae_Subgroup_3
Nitrospiraceae
Chitinophagaceae
SC_I_84
Hyphomicrobiaceae
Gemmatimonadaceae
Steroidobacteraceae
Nitrosomonadaceae
Reyranellaceae
Desulfarculaceae
Woeseiaceae
Blrii41
Gaiellaceae
Methyloligellaceae
Burkholderiaceae
Xanthobacteraceae
Dongiaceae
Phylum
original
cultivated
Genus
Phylum
0.6
0.4
0.2
0
-0.2
-0.4
-0.6
Acidobacteria
Actinobacteria
Bacteroidetes
Firmicutes
Fusobacteria
Nitrospirae
Proteobacteria
SWB02
Gaiella
Steroidobacter
MND1
Dongia
Bryobacter
Clostridium_sensu_stricto_1
JTB255_marine_benthic_group
Sphingomonas
Chryseolinea
Nitrospira
RB41
Aggregatibacter
Ellin6067
Candidatus_Solibacter
Altererythrobacter
Haliangium
Escherichia_Shigella
Reyranella
Acidibacter
Pedomicrobium
Neisseria
Enterococcus
Prevotella_7
Haemophilus
Lactobacillus
Lactococcus
Clostridium_sensu_stricto_12
Klebsiella
Pseudomonas
Veillonella
Pantoea
Fusobacterium
Streptococcus
Weissella
Phylum
original
cultivated
(B)
Family
Phylum
0.6
0.4
0.2
0
-0.2
-0.4
-0.6
Acidobacteria
Actinobacteria
Bacteroidetes
Firmicutes
Fusobacteria
Gemmatimonadetes
Nitrospirae
Proteobacteria
Verrucomicrobia
Enterobacteriaceae
Prevotellaceae
Streptococcaceae
Fusobacteriaceae
Lactobacillaceae
Pasteurellaceae
Neisseriaceae
Veillonellaceae
Unknown_Family
Acidothermaceae
Nitrospiraceae
o__Bacillales;f__Family_XI
Polyangiaceae
Koribacteraceae
Micropepsaceae
Chitinophagaceae
Xanthobacteraceae
Solibacteraceae_Subgroup_3
Chthoniobacteraceae
Haliangiaceae
Rhodanobacteraceae
Acetobacteraceae
Caulobacteraceae
Reyranellaceae
o__Clostridiales;f__Family_XI
Gemmatimonadaceae
Acidobacteriaceae_Subgroup_1
SC_I_84
Desulfarculaceae
Clostridiaceae_1
Pedosphaeraceae
Burkholderiaceae
Nitrosomonadaceae
Sphingomonadaceae
Micrococcaceae
Phylum
original
cultivated
Genus
Phylum
0.6
0.4
0.2
0
-0.2
-0.4
-0.6
Acidobacteria
Actinobacteria
Bacteroidetes
Firmicutes
Fusobacteria
Gemmatimonadetes
Nitrospirae
Proteobacteria
Verrucomicrobia
Lactobacillus
Neisseria
Fusobacterium
Haemophilus
Lactococcus
Veillonella
Klebsiella
Prevotella_7
Prevotella
Rothia
Sphingomonas
Pajaroellobacter
GOUTA6
Nitrospira
Acidothermus
Edaphobacter
Candidatus_Koribacter
Finegoldia
Gemmatimonas
Ellin6067
Candidatus_Udaeobacter
Escherichia_Shigella
Cupriavidus
Haliangium
MND1
Candidatus_Solibacter
ADurbBin063_1
Clostridium_sensu_stricto_1
Gemella
Reyranella
Bryobacter
Streptococcus
Bradyrhizobium
Acidibacter
Burkholderia_Caballeronia_Paraburkholderia
Phylum
original
cultivated

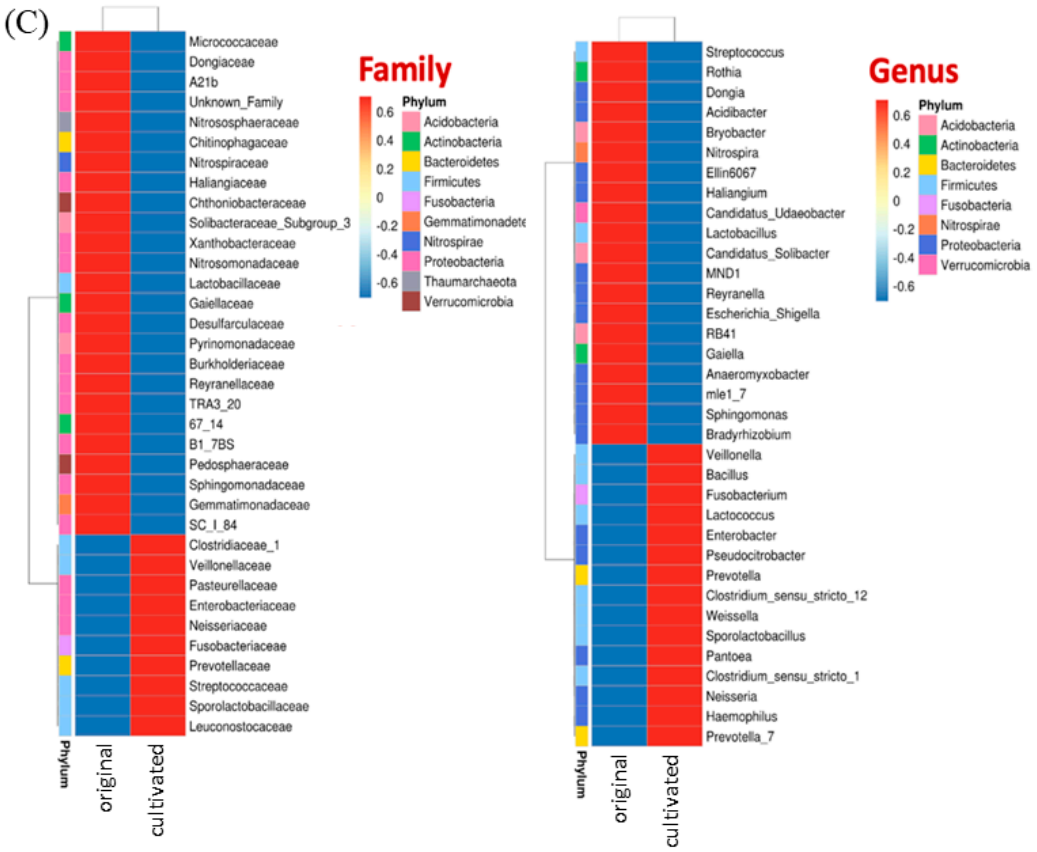

Supplementary Figure S6. The main biomarkers for (A) irrigation water, (B) soil, and (C) rhizosphere soil in original samples compared to the cultured-samples.
